# The Environmental and Ecological Benefits of Edible Insects: A Review

**DOI:** 10.1002/fsn3.70459

**Published:** 2025-06-20

**Authors:** Belay Gezahegn Gebreyes, Tilahun A. Teka

**Affiliations:** ^1^ Department of Postharvest Management Jimma University College of Agriculture and Veterinary Medicine Jimma Ethiopia; ^2^ Ethiopian Institute of Agricultural Research Teppi Agricultural Research Center Teppi Ethiopia

**Keywords:** alternative protein source, edible insects, environmental benefit, food chain, health benefit

## Abstract

Proteins are essential for human health, traditionally sourced from animal products like meat, eggs, and fish, or plant‐based alternatives such as legumes. In regions like sub‐Saharan Africa, where resources are limited, protein intake is often a challenge. With the global population rising rapidly and traditional protein sources under strain, innovative and sustainable alternatives are needed. Edible insects, once overlooked, are now recognized as a promising solution due to their high nutritional value and environmental benefits. Consumed globally in various cultures, insects provide a nutritious, unconventional protein source with significantly lower environmental impacts than conventional livestock, aligning with sustainability goals. This review examines the nutrient composition of edible insects, focusing on their protein, fat, amino acids, mineral content, and digestibility. It also highlights the role of insects as pollinators and contributors to biodiversity, emphasizing their importance in supporting agriculture. Additionally, the review explores processing techniques for developing insect‐based products. The nutritional value of edible insects varies based on the substrate used for rearing. By understanding insects' potential in human nutrition, we can address challenges posed by population growth while promoting a sustainable, eco‐friendly food system that benefits both human health and the environment.

## Graphical abstract

Edible insects offer a sustainable and nutrient‐rich alternative to traditional protein sources, with significantly lower environmental impacts. This review highlights the nutritional composition of various insect species and explores their role in biodiversity, agricultural support, and food system sustainability, emphasizing their potential to address global protein challenges.

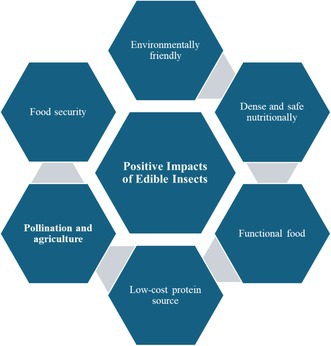

## 1 Introduction

By 2050, the global population is projected to reach 9.7 billion (Colgrave et al. 2021), with food demand estimated to rise by 70% (FAO 2009). To meet these demands, there is a necessity for alternative protein sources that transcend traditional options, such as meat, fish, poultry, dairy, aquaculture, and legumes. In 113 nations, insect consumption is a conventional practice, with approximately 2000 edible species identified, including beetles, caterpillars, bees, wasps, ants, grasshoppers, locusts, crickets, and cicadas (Kouřimská and Adámková 2016). Regions such as Africa, Asia, and Latin America widely embrace entomophagy, as defined by Rodríguez et al. (2019), as the utilization of insects for human nutrition.

Incorporating insects into diets can significantly enhance global food security, particularly in financially constrained countries that address hunger (Jantzen et al. 2019). Insects are a promising protein source that offers economic, environmental, and dietary advantages (Ordonez‐Araque and Egas‐Montenegro 2021; Rodríguez et al. 2019). Studies suggest that insects represent potential protein sources and substitutes for other proteins, with insect protein concentrates having the potential to replace traditional sources in food formulations (Turnagöl et al. 2022).

Insects provide a substantial protein basis, offering all the essential amino acids for human nutrition throughout their developmental phases (Churchward‐Venne et al. 2018; Tang et al. 2019). Entomophagy ensures sufficient protein, essential fats, and fiber for food security without significant financial costs, potentially alleviating global malnutrition and hunger (Sanchez‐Mártín et al. 2018). The nutritional composition of edible insects is significantly influenced by the type and quality of the substrate they are reared on, which directly affects their protein, fat, and micronutrient content (Riekkinen et al. 2022).

Edible insects not only offer nutritional benefits but also positively affect the environment (Lange and Nakamura 2021; Matandirotya et al. 2022). They are a unique and highly nutritious protein source consumed globally (Tao and Li 2018; Smith et al. 2021). Importantly, insect consumption is environmentally friendly, requires fewer resources, and emits fewer greenhouse gases than traditional livestock farming does (Lange and Nakamura 2023; Jankielsohn 2023; Oonincx et al. 2010). This aligns with the urgent need for sustainable food production. Additionally, our comprehensive review delves into the nutritional content of various edible insect species, emphasizing their role as pollinators, contributors to biodiversity, and agricultural support (Silva et al. 2020; Jankielsohn 2023; Lange and Nakamura 2023).

In conclusion, edible insects exhibit superior protein levels and production efficiency compared to traditional foods (Kim et al. 2019). This review highlights the potential of edible insects as a protein source, offering a sustainable alternative to meet the rising protein demand driven by factors such as climate change, population growth, and escalating costs associated with traditional protein‐rich options. Edible insects not only provide nutritional benefits, but also contribute positively to the environment, aligning with the imperative for sustainability in food production systems.

## 2 Nutritional Composition of Edible Insects

An estimated 2 billion individuals across regions such as Africa, Asia, Central and South America, and Australia currently incorporate insects into their diets for both their appealing taste and cost‐effective, nutritionally rich attributes. Globally, there are approximately 1 million insect species cataloged, of which approximately 1500–2000 are recognized as suitable for human consumption (Akhtar and Isman 2018). The most commonly consumed insect categories worldwide include beetles (Coleoptera, 31%), caterpillars (Lepidoptera, 18%), bees, wasps, and ants (Hymenoptera, 14%); grasshoppers, locusts, and crickets (Orthoptera, 13%); cicadas, leafhoppers, planthoppers, scale insects, and true bugs (Hemiptera, 10%); termites (Isoptera, 3%); dragonflies (Odonata, 3%); flies (Diptera, 2%); and other miscellaneous species (5%) (van Huis et al. 2013). Most insect species offer valuable minerals, vitamins, fatty acids, proteins, and fibers, making them a promising source of essential nutrients.

### 2.1 Protein Contents of Edible Insects

Insects can serve as beneficial alternative protein and nutrient sources for people residing in both developed and underdeveloped regions (Srivastava et al. 2009; Verkerk et al. 2007). Although their amino acid composition is generally like that of traditional meats, edible insects typically contain a higher proportion of crude protein. They are typically considered to be 76%–96% edible for human consumption and can provide essential amino acids in optimal quantities (Belluco et al. 2015; Nowak et al. 2016; Payne et al. 2016). The crude protein content on a dry weight basis typically falls within the range of 40%–75%, depending on the species, diet, type, and developmental stage (Tang et al. 2019). Chemical analysis of edible insects has revealed significant nutrient diversity, which is influenced by species, diet, type, and stage of development (Oonincx and Van Der Poel 2011; Kouřimská and Adámková 2016).

The way food is prepared before consumption (boiling, frying, baking, or drying) is also likely to have an impact on nutritional composition. Despite variations in their nutritional makeup, insects are often regarded as an important source of food for human consumption. Insects are a great source of protein for the human diet because they are not only healthy and delicious, but also environmentally sustainable (Akhtar and Isman 2018). Proteins play a vital role in our diets, as they provide essential and nonessential amino acids. As the human body lacks the ability to synthesize essential amino acids (EAAs) on its own, it is imperative to acquire them from food intake.

Animals and plants both contain proteins; however, due to insufficient and/or imbalanced concentrations of essential amino acids (EAAs), plant proteins have a lower nutritional value than animal proteins (Millward et al. 2008). In contrast to commonly consumed animal meats such as beef, pork, chicken, and lamb, various insects such as termites, grasshoppers, caterpillars, weevils, and houseflies offer superior protein content, as highlighted by Srivastava et al. (2009). The protein content of an insect is a critical factor for evaluating the significance of insects in entomophagy. Generally, edible insects exhibit elevated protein levels and serve as viable alternative protein sources, particularly those belonging to the order Orthoptera, which includes grasshoppers, crickets, and locusts.

Several studies, including those conducted by Xiaoming et al. (2010) and Ramos‐Elorduy et al. (2007), have documented that various grasshopper species and numerous species within the order Hymenoptera exhibit protein levels as high as 77% when measured on a dry‐matter basis (Table 3). The total protein composition of seven different insect species ranges from 38.4% to 62.2% based on dry matter (Bednářová et al. 2013). In a study conducted by Meyer‐Rochow et al. (2021), factors such as species, developmental stage, sex, dietary choices, and processing methods influenced the chemical composition, nutritional quality, sensory attributes, and overall acceptability of edible insects. The protein levels in most edible insects studied were like those found in beef, fish, or shrimp. However, in specific instances, such as chapulines (a type of grasshopper from the Sphenarium genus) commonly consumed in various preparations in Mexico, palmworm beetles, adult termites, adult locusts, and grasshoppers, the protein content may be even higher, as reported by the FAO (2012) (Table 3).

### 2.2 Fat Contents of Edible Insects

Additionally, edible insects represent a promising source of nutrition, and their fat content is a key area of research (Kouřimská and Adámková 2016). These insects exhibit a diverse array of healthy fats, including monounsaturated and polyunsaturated fats (Kolobe et al. 2023). Insect fats provide the body with a significant source of energy and essential fatty acids. Specific fatty acids such as linoleic acid and α‐linolenic acid play a vital role in sustaining human health (Paul et al. 2017). Insects have up to 43% of their dry weight in fat, making it the second most essential nutrient in the body (Zhou et al. 2022). Fat content varies among species, life stages, and preparation methods, offering a versatile range of dietary options (Tang et al. 2019; Hlongwane et al. 2022). The lipid profiles of edible insects contribute to their overall energy density, making them a concentrated source of calories (Hlongwane et al. 2022; Lange and Nakamura 2021). As sustainable and protein‐rich alternatives to traditional livestock, edible insects are gaining attention because of their potential to address global food security challenges (Lange and Nakamura 2021).

The composition of saturated fat (SFA), monounsaturated fatty acids (MUFA), and polyunsaturated fatty acids (PUFA) in insect fat determines its nutritional quality (Kolobe et al. 2023). The composition of dietary fatty acids has been linked to diseases such as cancer, diabetes, and cardiovascular diseases. Unsaturated fatty acids are significantly associated with human health and disease (Kolobe et al. 2023; Mirmiran et al. 2020). The majority of edible insect fat is high in unsaturated fatty acids, which are highly advantageous to humans (Kolobe et al. 2023). Research indicates that insects share a comparable fatty acid composition with fish and chicken, but they have a higher concentration of unsaturated fatty acids, particularly polyunsaturated fatty acids (Khalifah et al. 2023; Kuntadi et al. 2018; Rettore et al. 2016). Further exploration of edible insect fat composition and nutritional benefits is crucial to unlock the full potential of these eco‐friendly and nutrient‐dense food sources.

### 2.3 Vitamin Contents of Edible Insects

They also contain riboflavin, pantothenic acid, biotin, and, in certain instances, folic acid, although there is notable variability in the data (Kouřimská and Adámková 2016). The amount of vitamin B12 found in crickets and cockroach nymphs was high (11 times that of beef, 7 times that of salmon, and 59 times that of chicken). Termites and crickets are two other insects that are excellent sources of iron and zinc. Most insects that can be eaten are rich in nutrients, particularly vitamins B1 (thiamine) and B2 (riboflavin), as noted by Bukkens (2005). Compared to beef, edible insects boast significantly higher nutritional values, including three times the iron, 2.5 times the vitamin B12, and almost 15 times the magnesium content. The magnesium and zinc contents of soldier fly larvae are twice as high as those of beef, and they contain 71 times more calcium (Bukkens 2005; Akhtar and Isman 2018). Edible insects supply 15 times more thiamin, 9 times more riboflavin, and 2.6 times more vitamin B12 than beef, as reported by Bukkens (2005) and Akhtar and Isman (2018).

### 2.4 Fiber Contents of Edible Insects

Edible insects are increasingly recognized not only for their protein and micronutrient content but also for their dietary fiber, primarily in the form of chitin, a structural polysaccharide found in their exoskeletons. Unlike plant‐based fibers, chitin and its derivatives (such as chitosan) exhibit unique health‐promoting properties, including prebiotic effects, antioxidant activity, and potential roles in improving gut health and lowering cholesterol levels (Kipkoech 2023; Chakravorty et al. 2024; Stull and Weir 2023). The fiber content in insects varies widely depending on species, developmental stage, and processing method, ranging from 1.01% to over 12.56% of dry matter in some cases (Zhou et al. 2022). While not all chitin is digestible by humans, partial fermentation in the colon may still provide beneficial effects like insoluble dietary fiber (Kipkoech 2023). As such, edible insects offer a promising and underexplored source of functional dietary fiber that could complement traditional fiber sources in human diets (Zhou et al. 2022).

### 2.5 Mineral Contents of Edible Insects

Edible insects have emerged as a sustainable and nutrient‐rich food source, with the potential to provide several essential nutrients, including crucial minerals that are vital for human health (Zhou et al. 2022). This section aims to delve into the mineral composition of edible insects, offering insights into their nutritional significance beyond their commendable protein content. For example, edible insects such as crickets, mealworms, and grasshoppers are recognized for their substantial protein content (Zhou et al. 2022; Zielińska et al. 2015; Mabelebele et al. 2023). However, their nutritional profile extends far beyond the protein profile, and they constitute minerals. In general, minerals are categorized into macroelements, which are required in substantial quantities (such as Ca, Mg, K, and Na), and trace elements (including Fe, Cu, Zn, and Mn), which are required in smaller amounts (Ali 2023; Meyer‐Rochow et al. 2021). In the context of food safety, minerals can be classified into essential elements (such as Ca, Mg, Fe, Zn, and K) and nonessential elements encompassing heavy metals (e.g., Hg, Pb, Cd, Cr, and As), which may pose harm when present in excessive concentrations (Nag et al. 2022; Ali 2023; Meyer‐Rochow et al. 2021). The essential mineral content of an edible insect is derived from the feed it eats, insect species, and insect growth phase (Lu et al. 2024; Zhou et al. 2022; Meyer‐Rochow et al. 2021). However, it is crucial to know that the feed they eat might also contain harmful heavy metals that insects can absorb (Malematja et al. 2023). In addition, edible insects may accumulate heavy metals, dioxins, and other potentially harmful substances as a result of harvesting and processing (Lu et al. 2024; Zhou et al. 2022; Meyer‐Rochow et al. 2021).

Several edible insects showed substantial amounts of micronutrients, including copper, iron, magnesium, manganese, phosphorus, selenium, and zinc (Kouřimská and Adámková 2016). The iron content in most edible insects is equivalent to or exceeds that of bees, as reported by Rumpold and Schl (2013). Numerous insects, including grasshoppers, ants, caterpillars, and crickets, have extremely high calcium contents. Iron, zinc, calcium, magnesium, and niacin (vitamin B3) were highly concentrated in houseflies. The findings of Bukkens (2005) and Akhtar and Isman (2018) highlighted that the larvae of black soldier flies serve as a valuable dietary source of calcium, iron, zinc, potassium, niacin, and vitamin B12. High concentrations of phosphorus (604.66 ± 4.11 mg/100 g) and potassium (897.83 ± 1.55 mg/100 g). The moderately present elements were magnesium (109 ± 0.82 mg/100 g), calcium (213.66 ± 3.86 mg/100 g), and sodium (246.66 ± 2.62 mg/100 g). The zinc concentration was 8.24 ± 0.047 mg/100 g, while the iron concentration was 5.51 ± 0.45 mg/100 g in cricket (*Coiblemmus compactus*) samples (Lokeshkumar et al. 2022).

The mineral content of termites of the genus *Macrotermes* ranges from 0.14–114, 0.21–15, 0.03–5, 0.08–714, and 0.15–81 mg/100 g of Fe, Zn, Cu, Mn, and Mg, respectively (Oibiokpa 2017; Verspoor et al. 2020). Consequently, incorporating edible insects into one's diet alongside other food sources rich in essential minerals could enhance the overall nutrient intake, as suggested by Bernanrd and Womeni (2017). In summary, the nutritional profile of edible insects includes minerals, vitamins, proteins (with specific amino acid compositions), fats, dietary fiber, and energy content. This nutritional composition can vary based on factors such as the insect's origin, developmental stage, diet, and environmental conditions (e.g., temperature and humidity), as explored by Finke and Oonincx (2014).

## 3 The Effects of Substrate on the Nutritional Composition of Edible Insects

The exploration of edible insects as sustainable and nutritious food sources has gained significant attention in recent years. One crucial aspect of this investigation is the understanding of the effects of the substrate, the material on which the insects are reared, and their nutritional composition (Pinotti and Ottoboni 2021). The substrate, or material on which insects are reared, plays a pivotal role in shaping their nutritional content. Several studies have underscored the significance of substrates in influencing the protein (Fuso et al. 2021), fat, and amino acid content of edible insects. The type of substrate not only affects insect growth and development, but also leaves a distinct imprint on their nutritional composition, making it a crucial factor in optimizing their utility as a food source (Riekkinen et al. 2022).

### 3.1 Types of Substrates Used in Edible Insect Farming

The nutritional profile of edible insects is significantly influenced by the type of substrate or feed on which they are reared. Understanding these effects is essential for optimizing insect farming for human consumption. The main types of substrates commonly used in insect farming include the following:

#### 3.1.1 Agricultural By‐Products and Plant‐Based Substrates

Many edible insects are reared on agricultural residues such as crop leaves, husks, and bran. For example, mealworms ( *Tenebrio molitor* ) fed on wheat bran exhibit high protein and fat content, but the exact composition varies with substrate quality (Musembi et al. 2024). Similarly, crickets ( *Acheta domesticus* ) grown on plant‐based substrates show differences in amino acid profiles depending on the nutrient content of the feed (van Huis 2020). These substrates are sustainable and contribute to the circular bioeconomy by valorizing waste streams (Madau et al. 2020).

#### 3.1.2 Food Waste and Organic Waste Substrates

Feeding edible insects on organic food waste offers an eco‐friendly method to convert waste into protein‐rich biomass. Black soldier fly larvae ( *Hermetia illucens* ) reared on fruit and vegetable waste have demonstrated increased fat content and altered fatty acid profiles compared to those fed on standard diets (Camperio et al. 2025; Tepper et al. 2024; Mannaa et al. 2024). However, substrate contamination and safety issues must be carefully managed to prevent the accumulation of harmful substances (Alejandro Ruiz et al. 2025).

#### 3.1.3 Animal‐Based and Mixed Substrates

Some edible insects can be fed substrates containing animal by‐products or mixed materials, which may enhance certain nutrients. For instance, housefly larvae grown on poultry manure show increased mineral content, including calcium and phosphorus, compared to plant‐only substrates (Hussein et al. 2017; Kullan et al. 2025; Zou et al. 2024). Mixed substrates often improve growth rates and nutrient density but raise questions about sustainability and feed safety (Kolobe et al. 2024).

#### 3.1.4 Synthetic and Formulated Diets

To standardize nutritional output, formulated diets with controlled nutrient profiles have been used in research settings. These diets allow precise manipulation of macronutrients and micronutrients, enabling studies on how specific components affect insect nutritional composition. For example, protein‐enriched synthetic diets have been shown to increase the essential amino acid content in crickets (Pinotti and Ottoboni 2021).

There are studies that can prove the effects of substrate on the nutritional composition of edible insects: One of the key observations of these studies is that there is a direct correlation between substrate composition and the protein content in edible insects (Queiroz et al. 2023). For instance, insects reared on protein‐rich substrates exhibit higher protein levels than those reared on carbohydrate‐based substrates (Kröncke and Benning 2023). This insight holds immense promise for industries seeking to harness insects as a protein source, as it suggests that manipulating the substrate can be a strategic tool to enhance the nutritional value of the end product (Riekkinen et al. 2022).

Moreover, the lipid or fat content of edible insects is intricately linked to the substrate on which they are raised (Riekkinen et al. 2022). Research by Riekkinen et al. (2022) and Kolobe et al. (2023) revealed that variations in substrate composition can lead to significant differences in fatty acids, such as omega‐3 (Oonincx et al. 2020) and omega‐6, contributing to the overall lipid content (Riekkinen et al. 2022; Rutaro et al. 2018). This finding is particularly relevant, as it opens avenues for tailoring insect‐derived fats to meet specific nutritional requirements, addressing the diverse needs of consumers and industries alike (Zhou et al. 2022).

Amino acids, the building blocks of proteins, are another focal point for understanding the impact of substrates on insect nutrition. Study (Riekkinen et al. 2022) highlighted the role of substrates in determining the amino acid composition of edible insects. This insight is invaluable for formulating insect‐based products that can serve as complete and balanced sources of essential amino acids, which is a crucial consideration for meeting dietary needs (Queiroz et al. 2023).

Micronutrient content, including vitamins and minerals, also varies depending on the substrate. Insects reared on substrates rich in certain vitamins and minerals exhibit enhanced nutritional value (Riekkinen et al. 2022; Lu et al. 2024). This aspect is particularly crucial for addressing micronutrient deficiencies prevalent in certain populations.

The impact of substrates extends beyond basic nutritional components to bioactive compounds with potential health benefits (Ros‐Bar et al. 2022). Edible insects reared on specific substrates may contain higher levels of bioactive compounds such as antioxidants, antimicrobial peptides, and chitin (Rivas‐Navia et al. 2023; Schwarz and Zarei 2023). These compounds not only contribute to the physiological functions of insects but also hold promise for potential health‐promoting effects in consumers (Li et al. 2023).

In summary, the nutritional value of edible insects is closely linked to the environment in which they are grown, particularly the substrate they consume. Addressing the global challenges in food security and sustainable protein sources requires a focus on understanding and controlling these substrates. This brief overview highlights exciting research on the relationship between substrates and the nutritional content of edible insects, offering the potential for innovative and sustainable solutions from alternative protein sources. This knowledge is essential for both large‐scale insect production and smaller farming projects to secure the economic viability and ecological sustainability of insect‐based food production.

### 3.2 Consumable Insects for Human Dietary Use

The integration of insects into human food and animal feed products, along with their potential for marketing in well‐established and emerging markets, has been suggested. Advocates contend that insects provide a notably more environmentally friendly substitute for traditional animal protein sources, potentially mitigating environmental challenges stemming from the dietary needs of the expanding global population (Guiné et al. 2021). To secure food security, national governments and assistance programs should pay more attention to the production of edible insects because they have economic, nutritional, and ecological benefits. The year‐round availability of insects can be achieved by creating improved conservation techniques (Selaledi et al. 2021).

Beetles, caterpillars, bees, ants, crickets, grasshoppers, and locusts rank among insects with the highest levels of consumption (Raheem et al. 2018). Consumable insects are of considerable importance (Mutungi et al. 2017). In Africa, a wide variety of insects, specifically 470 different species, are a part of the diet, as highlighted in their (Kelemu et al. 2015) study. This rich diversity in insect consumption reflects the region's culinary practices and highlights the importance of insects as a source of nutrition and sustenance in African cultures (Kelemu et al. 2015). Different techniques have been employed to process various types of insects for human consumption. In every instance, insects were taken from their natural wild habitats. The intestines, wings, legs, and/or heads were removed based on the specific species and washed in cold or lukewarm water to rinse and separate the extraneous material and unappealing components.

After sun‐drying, the insects were processed into powder, roasted, boiled, and consumed. In some cases, insects are consumed raw (Mutungi et al. 2017). Consumable insects are widely accessible in school lounges and outdoor marketplaces in several African countries, such as Zimbabwe, Zambia, and Nigeria, and they have grown into a lucrative industry (Mutungi et al. 2017). Thus, cleaning is the first step in the basic processing of insect‐ and insect‐based foods on the African continent.

This is achieved by employing either dry or wet thermal treatments, which can be optionally followed by a drying and grinding process prior to packaging and storage, as outlined in the study by Mutungi et al. (2017).

### 3.3 Derivatives of Consumable Insects for Human Dietary Use

Edible insects offer versatility in processing and packaging, allowing for the creation of various products, including dried insects presented as either chunks or powder, suitable for incorporation into packaged food items, such as snacks, pasta, or baked goods, as discussed by Reverberi (2021). In a review article by Kwiri et al. (2014), the potential use of mopane worms (*Gonimbrasia belina*) as a protein source in fortified blended foods in Zimbabwe is highlighted.

In Kenya, research has been conducted on developing new protein‐enriched food products by combining wheat flour and edible termites and evaluating the product's nutritional and sensory qualities (Kinyuru et al. 2009). In Zimbabwe and the northern regions of South Africa, people consume stinkbugs ( *Encosternum delegorguei* ) either raw or after cooking. Typically, insects are prepared by removing their heads, and occasionally, they are fried and subsequently sun‐dried for preservation, as described in the study conducted by Musundire et al. (2016). The essential steps in the processing of edible insects involve initial cleaning, which may entail sieving or repeated washing with cold or warm water, followed by heat treatment options such as blanching, boiling, or roasting. Subsequently, the insects are subjected to drying, which can be achieved by either sun‐ or oven‐drying. Finally, they are either chopped into a granular form or ground into a paste, as outlined in the research by Mutungi et al. (2017).

The emergence of edible insects as food sources has expanded, resulting in the creation of insect‐derived components rather than finalized food products that maintain their original characteristics (Nongonierma and Fitzgerald 2017; Mishyna et al. 2018). Currently, there is a growing trend toward incorporating edible insect‐derived items into the food system, with efforts to counteract insect aversion in our culture by presenting insects as a direct food choice. Additionally, utilizing insects as ingredients in food production holds promise for establishing sustainable business models (Han et al. 2017). However, one of the major challenges faced by the edible insect industry is the lack of systematic efforts to ensure safety and prolong the shelf life (van Huis 2016).

Furthermore, standardization and quality control are needed for insect farming to use edible insect products for human food consumption as alternative sources of nutrition, as these products have high protein and other nutrients (Han et al. 2017). The following types of edible insect products are available globally: some of foods (salads, cookies, protein bars, bread, burger patties, crackers, chips, falafel, flours, granola, alternative meats, pasta, and noodles, sausage), beverages (beers, milk, milkshakes, soft drinks, spirits, and alcoholic beverages and drinks fortified with protein), confectionary (candy and lollipops, chocolates, ice creams, cookies), and other products (bitters, butter, oils, pesto, spices, and other products) (Agency 2021; Reverberi 2021; Skotnicka et al. 2021; Akhtar and Isman 2018); In conclusion, eating insects can be seen from two perspectives: first, as a source of protein for underdeveloped nations, and second, as an optional basis for protein for the more advanced nations globally (Skotnicka et al. 2021).

## 4 Processing Techniques for Edible Insects as Human Food

The demand for edible insects is growing rapidly, with an increasing requirement for novel products in various recognizable formats and as supplementary ingredients (Van Thielen et al. 2018). A review of the key technologies used is crucial in this case. According to Raheem et al. (2018), production procedures commence with the post‐harvesting of raw insects and end with the creation of edibles and waste, some of which are recovered and used as by‐products. Despite the enormous number of processes currently in use in the edible food industry, the majority can be categorized into a comparatively fewer number of operations with a similar fundamental philosophy and basically the same goals, based on the variety used and the ultimate product to be developed (Melgar‐Lalanne 2019).

Processing technologies for edible insects, along with their commercialization, employ a range of strategies, from traditional to innovative, offering a sustainable approach to enhance global food security (Melgar‐Lalanne 2019). In traditional cultures, insects undergo various processing methods, such as steaming, roasting, smoking, frying, stewing, and curing to improve their sensory attributes, nutritional qualities, and shelf life (Grabowski and Klein 2016; Li et al. 2023; Belhadj Slimen et al. 2023; Zhou et al. 2022; Liceaga 2022). New technologies have emerged to boost consumer interest by transforming insects into non‐recognizable forms like powders or flour (Tavares et al. 2022; van Huis and Rumpold 2023). These processes typically involve blanching to reduce microbial counts and deactivate enzymes (Adetoro et al. 2021; Ojha, Bekhit, et al. 2021). Techniques such as drying (sun‐drying, freeze‐drying, oven‐drying, fluidized bed drying, and microwave‐drying) (Hernández‐Álvarez et al. 2021) and advanced methods (ultrasound‐assisted extraction, cold atmospheric pressure plasma, and dry fractionation) focus primarily on extracting protein, fat, and/or chitin (Belwal et al. 2022; Melgar‐Lalanne 2019; Ojha, Bekhit, et al. 2021; Noyens et al. 2021) as shown in Tables 1 and 2.

**TABLE 1:** Processing techniques of edible insects.

Name of insects/species	Processing techniques	Main findings	References
*Dragonfly nymphs*, *C. limbatus* , *H. caschmirensis*, *O. smaragdina* , *P. bartheleymi*, *V. affinis indosinensis and O. fuscidentalis*	Roasting	Roasting edible insects decreased the microbial load compared to fresh ones in wild‐harvested insect samples	Kiewhuo et al. (2014)
*Eulepida mashona* (beetle) and *Henicus whellani* (cricket)	Boiling, roasting, or combined boiling and roasting	Boiling led to protein loss in both insects, while roasting did not lead to significant protein loss. Protein digestibility decreased with boiling, but there was no significant difference for beetles with roasting. Boiling and roasting had no significant impact on the iron and zinc content of both insects. Roasting is preferable to boiling, and in any case, a brief boiling period is recommended	Manditsera et al. (2019)
*Ruspolia differens* (longhorn grasshopper)	Freeze‐drying and oven‐drying	After blanching, both freeze and oven‐drying maintain proximate, fatty acid, and mineral composition. These drying methods are suitable for developing insect‐based food products without significant nutritional alterations	Fombong et al. (2017)
Adult locust and silkworm pupae	Freeze drying, oven drying, and microwave drying	Drying methods: freezing, oven drying, and microwave drying modified the odor and sensory characteristics of locusts and silkworms and, consequently, cookies made with these insect species. These factors should be considered in the processing of insects and the formulation of novel insect‐based foods	Mishyna et al. (2020)
Cricket species (wild *Scapsipedus icipe* and *Gryllus bimaculatus*)	boiling, sun‐drying, freeze‐drying, snap‐freezing and deep‐frying	Snap‐freezing, deep‐frying, and freeze‐drying were found to be the most effective processing techniques for lowering microbial counts	Gatheru et al. (2019)
*Hermetia illucens* larvae	blanching, roasting, and superheated steam blanching	This study employed three pre‐treatment techniques (blanching, roasting, and superheated steam blanching) to assess the drying speed and quality features of *H. illucens* larvae. When hot air‐drying *H. illucens* larvae, blanching and superheated steam blanching are thought to be the best pre‐treatment techniques for enhancing their processing qualities	Lee et al. (2023)

Insects species/name	Processing techniques	Main findings	References
Black soldier fly ( *Hermetia illucens* ), cotton leaf worm (*Spodoptera littoralis*), Adult grasshoppers (*Ruspolia differens*), Adult house crickets ( *Acheta domesticus* )	Boiling, toasting, solar‐drying, oven‐drying, (boiling + oven‐drying), (boiling + solar‐drying), (toasting + oven‐drying), (toasting + solar‐drying)	With species‐ and treatment‐specific patterns, the processing techniques improved microbiological safety and modifies nutritional values of the edible insects. In the following order: toasting > boiling > oven‐drying > solar‐drying, these processes decreased dry matter crude fat content by 0.8%–51%. In the same sequence, the protein content increased by 1.2%–22%	Nyangena et al. (2020)
Stinkbug (*Encosternum delgorguei*)	Toasting, microwave drying, oven drying, and sun drying	The nutritional composition, microbiological quality, and sensory attributes of processed edible stinkbugs were significantly (*p* < 0.05) influenced by various drying methods, including toasting, microwaving, oven drying, and sun drying	Dandadzi et al. (2023)
Black soldier fly ( *Hermetia illucens* )	Ultrasound‐assisted alkaline extraction black soldier fly larvae protein (BSFLP)	Ultrasound‐assisted alkaline extraction enhances the nutritional, structural, and functional properties of black soldier fly larvae protein (BSFLP)	Xu et al. (2023)
Black soldier fly (*Hermetica illucens*) and yellow mealworm ( *T. molitor* ) larvae	Cold atmospheric pressure plasma	The research demonstrated how nonthermal processing stages and extraction conditions affect proteins and the specific techno‐functional properties of insect intermediates. The purpose of this study was to anticipate their potential applications in food and feed processing	Bußler et al. (2016)
*Clanis bilineata* larvae	Ultrasound‐assisted aqueous extraction (UAAE)	The utilization of ultrasound‐assisted aqueous extraction (UAAE) emerges as a viable method for the mass production of Clanis bilineata oil, suggesting its potential as a functional oil resource. Moreover, the DPPH radical scavenging and β‐carotene bleaching tests revealed that UAAE oil exhibited superior antioxidant activities compared to Soxhlet extraction‐derived oil	Sun et al. (2018)
Mealworm larvae ( *Tenebrio molitor* )	Dry fractionation	The process of dry fractionation for mealworm larvae emerges as a practical substitute for conventional wet fractionation techniques commonly employed in the processing of edible insects. It is a viable alternative, providing distinct advantages and potential improvements in the fractionation process for these insect larvae	Purschke et al. (2018)

**TABLE 2:** Edible insects processing techniques' advantages and disadvantages.

Processing techniques	Advantages	Disadvantages	References
Sun‐drying/Solar dryer	It is inexpensive and simple to facilitate drying	Cyclical nature of solar radiation; uneven drying; potential contamination, due to temperature variations Rehydration Potential contamination from animals, wind, or dust Extended drying duration	Mujumdar (2014)
Freeze‐drying (lyophilization)	Increased nutritional content Maintain the quality of proteins Maintain materials susceptible to heat	High price Oxidation of lipids Poor output	Mujumdar (2014), Mohd Zaini et al. (2023)
Oven‐drying	Minimal drying time and maximum precision	Degradation of proteins	Mohd Zaini et al. (2023), Mujumdar (2014)
Fluidized bed dryer	Rapid heat and mass transfer rates	Materials that are sensitive to heat may be affected by high temperatures and high electrical power consumption	Mujumdar (2014)
Infrared radiation	Regular heating, rapid heat transmission, and cost‐effectiveness	Controlling in distance	Khampakool et al. (2020), Hernández‐Álvarez et al. (2021), Pan (2021)
Microwave dryer	‐Low ambient temperatures can be used for drying. ‐Short drying times and quick drying speeds. ‐Easy drying conditions to regulate	High cost of equipment acquisition Inhomogeneous drying Minimal depth of penetration Texture damage	Mujumdar (2014), Changrue and Raghavan (2006)
Vacuum dryer	Maintain substances that are susceptible to heat.	Low level of production	Mujumdar (2014), Mella et al. (2022), Niu et al. (2019)

Processing techniques	Advantages	Disadvantages	References
Heat pump drying	Gentler drying with high energy efficiency and lower protein degradation compared to oven‐drying	Expensive and challenging to install Poor performance in cold climates	Kivevele and Huan (2014), Kim et al. (2022)
Solar‐assisted drying	More hygienic than drying in the sun. Enhanced drying effectiveness when paired with alternative techniques: simple to dry and less energy consumption	Microbiological risks, more expenses, humidity levels below 60%, and drying efficiency are influenced by climatic conditions	Suresh et al. (2023), Yahya et al. (2023), Kim et al. (2022)
Pulsed electric field	Reduced treatment duration Avoidance of nutrients Microbiological safety	The initial cost is high No impact on spores or enzymes Applicable only to liquids Limited energy efficiency	Alles et al. (2020), Taha et al. (2022), Kim et al. (2022)
Osmotic dehydration	Enhancing quality and preventing it from declining Minimal energy expenses Maintains flavor; minimizes the enzymatic browning reaction	An increase in sweetness or salinity A reduction in acidity	Kim et al. (2022), Ahmed et al. (2016)
Cold atmospheric pressure plasma	Cold atmospheric pressure plasma technologies, with limited and regulated penetration, minimize product‐process interactions, ensuring maintained product quality	Limited penetration ability The initial cost is relatively high	Hertwig et al. (2018), Reema Khanikar et al. (2022), Ojha, Bekhit, et al. (2021)

Below are some examples of processing technologies for edible insects.

### 4.1 Blanching

Blanching is a crucial step in the processing of agricultural products because it may inactivate enzymes that result in unwanted changes. When a food is blanched, the heating process is stopped by submerging it in frost water after briefly immersing it in boiling water and then removing it (Xiao et al. 2014). To minimize the presence of microorganisms and inhibit the activity of enzymes that can lead to food spoilage and potential illnesses, pretreatment methods are frequently employed in the preparation of edible insects, a practice adopted across various scales of production, including industrial and artisanal settings.

However, blanching is inefficient at removing or even lowering the number of mesophilic bacterial spores. Instead, it drastically lowered the total counts of mesophilic bacteria, yeast, and molds. Additionally, blanching lowers the total number of psychrotrophic and lactic acid bacteria numbers (Vandeweyer et al. 2017). To optimize antimicrobial benefits while minimizing quality degradation, it is advisable to create a tailored blanching method for each insect species. Blanching mitigates the microbiological risks associated with the consumption of edible insects and can be combined with other techniques to mitigate bacterial spore contamination (Caparros Megido et al. 2017). However, it remains essential that these treatments preserve the nutritional content of food and other essential quality attributes, such as texture and color, to meet the expectations of both consumers and industrial processing requirements (Melgar‐Lalanne 2019).

### 4.2 Drying

Drying is the most employed technology for extending the shelf life of food products. When it comes to processing edible insects, a range of drying methods is available, encompassing traditional approaches like (roasting, frying, and sun‐drying) alongside more modern methods such as freeze‐drying and microwave‐assisted drying (Melgar‐Lalanne 2019). The drying process reduces the overall moisture content, consequently limiting the amount of water accessible for degradative processes, including enzymatic reactions and those instigated by spoilage microorganisms. There is a direct correlation between water activity (AW) and microbial proliferation, with many bacteria growing at an AW of 0.65. Microorganisms tend to exhibit sluggish growth under low AW conditions, but they can swiftly resume their growth when moisture conditions become favorable (Grabowski and Klein 2016, 2017).

Reducing the amount of free water significantly enhances the concentration of dry matter in food products while maintaining the integrity of the tissues and the overall physical characteristics of the food unchanged, as reported by Lamidi et al. (2019). The optimal methods for drying edible insects include sun drying, freeze drying, and oven drying. Conversely, for insect‐derived flours and powders, commonly employed drying techniques include oven drying, freeze‐drying, and unconventional methods. In Western nations, one of the prevailing strategies to promote the consumption of insects by humans involves drying and pulverizing easily identifiable insects into unrecognizable powders, a concept advanced by Menozzi et al. (2017). Furthermore, drying lengthens the shelf life of products during transportation and storage.

However, before eating insects, they should be warmed to kill any remaining bacteria, even though blanching and drying procedures were used. It has been discovered that boiling dried insects for 30 min is an effective method for getting rid of total bacteria, including Enterobacteraceae, Staphylococcus, Bacillus, yeasts, and molds (Grabowski and Klein 2016). Sun drying is the oldest and most commonly used drying method because it requires little energy and produces lighter products that are easier to transport (Azzollini et al. 2016).

According to certain studies, sun drying controls microorganism spoilage and minimizes some hazardous substances, such as neurotoxins, while simultaneously improving the overall nutritional content of the product through the deactivation of protease inhibitors (Niassy et al. 2016). Nevertheless, the primary drawback of sun‐drying is the reduced hygienic value of both the processing method and end product. Based on how it looks, the insect is assumed to be dehydrated. Given that this is not a domestic process, neither the moisture content nor the water activity was determined.

Because of the lax hygiene standards used during the process, dried insects could become infected if they came into contact with dirty air (Caparros Megido et al. 2018). Smoking is a heating and curing process. It is one of the most ancient techniques for preserving various types of meat. The unprocessed product was exposed to smoke from the wood pyrolysis. In the case of insects, smoking is performed in a dry environment, and the processes of drying and curing occur concurrently. Heat and enzymes work together to promote protein and lipid modifications during the process (Tiencheu et al. 2013). The method most frequently employed for analyzing the nutritional properties of insects in scientific settings is freeze‐drying.

A best‐quality product with outstanding dietary content and an elongated storage time is produced because of the decreased temperature utilized along with the ensuing water sublimation, which is ideal for investigation; however, it is expensive to expand or increase the scale to an industrial level. In Europe, freeze‐dried edible insects are commonly purchased. Comparing results from studies on the same insect species is a complex task, mainly because of the predominant emphasis on physicochemical characterization in research, which often lacks details about the specific conditions employed (Caparros Megido et al. 2017). Products that are oven‐dried are analogous to those that are freeze‐dried and have lower power input expenses, less oxidation of the lipids, and higher solubility of proteins (Kröncke et al. 2018; Lautenschläger et al. 2016; Fombong et al. 2017).

After 16 min (aw = 0.30), microwave‐assisted drying lowered water activity, which significantly reduced the number of microbes present. *T. molitor* L. was used to assess the viability of microwave drying, and the results showed that aw values less than 0.6 could be attained for the finished goods. After drying, the protein, fat, and ash levels of the fresh and blanched larvae showed very slight variations. The simple major loss compared to freeze‐drying was vitamin B12, and the browning index remained consistent over the course of 4 months of storage (Lenaerts et al. 2018).

However, further research is required to determine how to preserve the sensory attributes of insects after microwave drying (Vandeweyer et al. 2017). Various drying techniques were assessed to achieve moisture content below 7.0% and a water activity level of less than 0.6 for *T. molitor* L. larvae. These methods include freezing, fluidized‐bed drying, microwave drying, vacuum drying in a vacuum oven, and conventional heat‐drying on a rotating rack. Scientists have concluded that the protein, fat, and fiber contents are only slightly altered by each drying procedure (Kröncke et al. 2018).

Beyond the evident reduction in water content, the use of different drying processes has an impact on some quality criteria. The selected drying method and the technological circumstances used could affect the functional properties of proteins, lipid oxidation, and color. As the insect will either be ingested in its entirety, as a milled ingredient, or as the only ingredient, the chosen drying technology should take these factors into account. Given the significant amount of lipid oxidation and unsanitary conditions associated with sun drying, it should not be regarded as a major method. Owing to its ability to remove the same amounts of protein, fat, and chitin by freeze‐drying, oven drying is the favored commercial drying technique (Azzollini et al. 2016; Purschke et al. 2018; Melgar‐Lalanne 2019).

### 4.3 Ultrasound‐Assisted Extraction of Edible Insects

Ultrasound‐assisted extraction of edible insects involves utilizing ultrasonic waves to enhance the extraction process (Xu et al. 2023). This innovative technique aims to efficiently extract valuable components from insects, such as proteins (Ma et al. 2023), fats, and chitin. The application of ultrasound waves facilitates improved yields by disrupting cellular structures and promoting the release of the desired compounds (Ojha, Bußler, et al. 2021). This method has been recognized for its effectiveness in optimizing the extraction of essential nutrients from edible insects, contributing to the development of sustainable and nutritionally rich food products (Zhang et al. 2023).

### 4.4 Cold Atmospheric Pressure Plasma

The cold atmospheric pressure plasma method for processing edible insects is a novel approach to enhance the quality and safety of insect‐based products (Ojha, Bekhit, et al. 2021). In this method, low‐temperature plasma is utilized to treat edible insects, effectively eliminating potential contaminants and improving their shelf life (Hertwig et al. 2018). The cold atmospheric pressure plasma process is known for its ability to inactivate microorganisms and enhance the overall safety of edible insect‐derived products (Table 1). This innovative method contributes to ensuring the microbiological safety of insect‐based foods, aligning with the broader goal of advancing sustainable and secure food‐processing techniques (Reema Khanikar et al. 2022).

### 4.5 Dry Fractionation Method

The dry fractionation method for processing edible insects is an innovative technique for separating and obtaining specific components from insect biomass (Purschke et al. 2018; Sweers et al. 2022). As shown in Table 1, this process involves mechanical separation at low temperatures, allowing for the extraction of distinct fractions, such as proteins, fats, and chitin, without the use of solvents or excessive heat. To avoid harsh conditions, the dry fractionation method preserves the quality and functionality of the extracted components (Kim et al. 2022). This novel approach contributes to the sustainable utilization of edible insects and offers a way to harness their nutritional and functional attributes efficiently for various applications in the food industry (Noyens et al. 2021; Sweers et al. 2022).

## 5 The Health Benefits of Edible Insects

Beyond their nutritional richness and environmental sustainability, edible insects provide a variety of health‐promoting benefits attributed to both their nutrient density and bioactive constituents. They are rich sources of complete proteins, essential amino acids, unsaturated fatty acids, and key micronutrients such as vitamin B12, riboflavin, folate, iron, zinc, and calcium—nutrients that are vital for addressing malnutrition, especially in low‐resource settings (Sánchez‐Estrada et al. 2024; Nowakowski et al. 2022; Li et al. 2023). In addition to these, insects contain various functional compounds like bioactive peptides (Hall et al. 2020; Zieli'nska et al. 2018), chitin and its derivative chitosan (Malm and Liceaga 2021; Mohan et al. 2020), phenolic compounds (Nino et al. 2021; Baigts‐allende et al. 2021), and beneficial fatty acids (Jantzen et al. 2019). These compounds have been linked to a range of health benefits, including antioxidant, antihypertensive, anti‐inflammatory, antimicrobial, and immune‐modulatory activities (Di Mattia et al. 2019; Hall et al. 2018; Cito et al. 2017; Tang and Dai 2018; Malm and Liceaga 2021; Flores et al. 2020; Tang and Dai 2016), with a beneficial effect on human health. For example, proteins derived from species like *Spodoptera littoralis*, *Bombyx mori* , *Schistocerca gregaria*, and *Bombus terrestris* have demonstrated angiotensin‐converting enzyme (ACE) inhibitory properties, suggesting potential for blood pressure regulation (Vercruysse et al. 2005). Both in vitro and in vivo studies support the therapeutic potential of edible insects, reinforcing their role not only as alternative protein sources but also as functional foods with promising applications in health and disease prevention, as shown in Table 3.

**TABLE 3:** A summary of tables of studies and review papers on edible insects.

Name of insects	Stage/parts of insects	Country/place were studied	Objective of study	Research finding of the study	References
House crickets, lesser mealworms, and black soldier flies	Egg, larva, pupa, and adult	Netherlands	To enhance the omega‐3 fatty acid content of edible insects	Adding just 4% of n‐3 fatty acids increased these three species' n‐3 content by 10–20 times, lowering their n‐6/n‐3 ratios from 18–36 to 0.8–2.4. This demonstrates the value of incorporating n‐3 fatty acid sources into insect diets to enhance their nutritional quality	Oonincx et al. (2020)
*I. oyemensis*, *L. migratoria*, *A. mellifera* , *M. subhyalinus*, *Rdifferens*, *G. trivittata* , *R. phoenicis*, *Vespula* spp., and *G. africana* *is*	Larvae, pupae, and adults	D.R. Congo	The objective of this study was to investigate the biodiversity, perception, consumption, and accessibility, host plants, harvesting methods, and processing methods of edible insects	The research finding of this study shows that their seasonal availability varies from species to species and is associated with the availability of the host plants. Species, local expertise, and customs all influence harvesting, processing, and preservation methods	Ishara et al. (2023)
Cricket	Adult	USA	The objective of this work was to find peptides produced from the in vitro gastrointestinal digests of cricket protein hydrolysates that were antihypertensive, anti‐glycemic, and anti‐inflammatory	For the molecular docking investigations, three new peptides—YKPRP, PHGAP, and VGPPQ—were selected. With binding energies like captopril, PHGAP and VGPPQ showed a higher level of non‐covalent interactions with the enzyme active site residues. The findings of this study show that edible cricket peptides have bioactive properties, particularly as ACE inhibitors	Hall et al. (2020)
Mealworms, locusts and crickets	Larva and adult	Poland	The aim of this research was to investigate the impact of heat treatment on the antioxidant and anti‐inflammatory characteristics of peptide fractions from hydrolysates generated through in vitro gastrointestinal digestion of edible insects	The application of heat treatment boosts the antioxidant and anti‐inflammatory attributes of peptides. Every peptide chosen and synthesized from insect proteins demonstrated both anti‐inflammatory and antioxidant properties	Zieli'nska et al. (2018)
Cricket Species	Adult	USA	To investigate the physicochemical characteristics of chitosan and explore its use as a hypolipidemic and antimicrobial agent obtained from two distinct cricket species.	This study's findings indicated that the food and pharmaceutical industries could benefit from using by‐products from the processing of insect proteins, such as chitin and chitosan.	Malm and Liceaga (2021)
Lepidoptera, Coleoptera, Orthoptera, Hymenoptera, Diptera, Hemiptera, Dictyoptera, and Odonata	All stages of insects	India	Examining present resources, methods of extraction, and the physicochemical characteristics of chitin and chitosan from insects	The findings from this review paper offer a more comprehensive understanding of how chitin derived from insect waste can be converted into valuable products, serving as an alternative chitin source to address concerns related to food security	Mohan et al. (2020)
Butterfly	Adult, wings	Colombia	To evaluate insects' phenolic compounds and their potential health advantages	Research conducted both in laboratory settings (in vitro) and within living organisms (in vivo) has revealed that bioactive compounds possess antioxidative, anti‐inflammatory, anticancer, and antibacterial attributes	Nino et al. (2021)
*A. cordifera B. mellifica & H. illucens*	Larvae	Mexico	To examine the structural and functional properties of protein extracts derived from consumable insects	According to the study's findings, emulsions made with insect protein from *A. cordifera* and *B. mellifica* were more stable than those made with *H. illucens* , which had larger globule sizes and quicker phase separation	Baigts‐allende et al. (2021)
Common edible insects	Information includes all stages in	54 African countries	To evaluate Africans' indigenous knowledge‐based consumption of edible insects to provide insights for research and policymaking	This study found that sun drying was the most widely utilized processing method as it required less input than frying, boiling, or roasting. The dried insects were preserved using chemicals like salt, palm oil, or pure honey to extend their shelf life. The study's results are discussed from the perspectives of science, policy, and business	Niassy et al. (2016)
Most common edible insects	Information includes all stages in	Canada	To explore the viability of insects as a protein source for human dietary needs	This review article demonstrated how different edible insects could serve as a protein source suitable for human consumption	Akhtar and Isman (2018)
Yellow mealworm ( *Tenebrio molitor* )	Larvae	Italy	To investigate how the drying process's kinetics and hygroscopic properties impact the nutritional value of yellow mealworms	Blanching caused notable changes in the adsorption isotherms and elevated the moisture content of fresh larvae while leaving their proximate composition unaffected. More color deterioration was achieved after rehydrating after lyophilization, most likely because of increased enzyme activity	Azzollini et al. (2016)
Most common edible insects	Information includes all stages in	Africa	To review postharvest processes and techniques and their effects on nutrition, safety, and the development of new edible insect products in the African context	This review contributed by assessing the information that is currently available on the postharvest procedures designed for consumable insects in Africa with the goal of finding areas that require investigate to be sparked	Mutungi et al. (2017)
Mealworms ( *Tenebrio molitor* )	Adult	Belgium	The objective of this research was to evaluate how various cooking techniques employed in households influence the nutritional content and microbial presence of mealworms	According to this study's findings, boiling and frying mealworms in a vacuum be the main effective ways to lower the level of microorganisms even if preserving their maximum protein and polyunsaturated fatty acid content	Caparros Megido et al. (2018)
Mealworms ( *Tenebrio molitor* ) and house crickets ( *Acheta domesticus* )	Adult	Belgium	The aim of this research is to assess the microbial safety of edible insects	The study results indicate that freeze‐drying and sterilization are efficient methods for decreasing the total aerobic bacterial count. This underscores the importance of cooking both fresh and smoked insects from non‐European origins before consumption. Importantly, the initial aerobic bacterial count in all untreated insect samples exceeded the limit set for fresh minced beef (6.7 log cfu/g)	Caparros Megido et al. (2017)
Longhorn grasshopper	Adult	Uganda and Kenya	To evaluate how freeze‐drying and oven drying, after blanching, impact the nutritional composition of longhorn grasshoppers	The study's findings demonstrated that both drying methods were successfully applied to develop insect‐based foods without significant nutritional changes	Fombong et al. (2017)
Most common edible insects	Information includes all stages in	Zimbabwe	To investigate edible insects' taste, nutritional value, availability, and consumption patterns in rural as well as municipal areas of Zimbabwe	The consumption of insects is impacted by the accessibility of edible insects, with 64.0% of urban participants and 83.0% of rural respondents being influenced by this factor. The potential contribution of insects to food security in urban environments may be there hampered via the reduced consumption of insect species in these locations. Hence, the marketing of entomophagy and the preservation of traditional insect processing skills should focus on urban consumers by offering delectable goods and emphasizing their nutritious worth	Manditsera et al. (2018)
Most common edible insects	Information includes all stages in	Spain	To explore the advantages of consuming edible insects for the well‐being of both humans and the environment	This study reveals how edible insects have health benefits by being an alternative resource of protein for human being as well as animal nourishment; in addition, they have a positive effect on the environment. More research into edible insects is critical for both human health and the environment	Ros‐Bar et al. (2022)
Mealworms	Adult	Netherlands	To research the environmental effects of using mealworms as a source of human protein	According to this study, mealworms be supposed to be there regarded as a further sustainable resource of fit for human consumption protein. When compared to chickens, pigs, and cattle as a source of human protein, mealworms emit significantly less greenhouse gases and require a lot less space	Oonincx and De Boer (2012)
Caterpillar (*Imbrasia epimethea*)	Larvae	Angola	This study's objective was to evaluate the impacts of various treatments on the nutritional composition of the edible caterpillar	The result of this research revealed that the nutritional composition of the caterpillars was relatively unaffected by the various preparation techniques	Lautenschläger et al. (2016)
Most common edible insects	Information includes all stages in	Sweden and Denmark	The goal of this review was to identify the primary factors that affect how consumers perceive and embrace insect‐based foods	In this review article, it was observed that the intricate emotional aspects, including feelings of disgust and neophobia, along with familiar flavors, textures, and situations, played a substantial role in shaping consumers' perceptions of insects as a food source and their willingness to accept them as such	Wendin and Nyberg (2021)
Most common edible insects	All stages of edible insects	South Korea	The aim of this work was to explore the current state and difficulties facing the industry for edible insects in the food, material, animal feed, and pharmaceutical markets, which are discussed in the paper	According to this study, the absence of high production capabilities and the engagement of bigger food firms are the main obstacles. To overcome these challenges, the following possible solutions, such as mass farming, manufacturing, and processing of consumable insects, can help the to solve the problems	Han et al. (2017)
Most common edible insects	All stages of edible insects	Mexico	To examine the health advantages and safety considerations of consuming edible insects, in addition to their nutritional value for humans	Because of their great nutritional composition as well as environmentally friendly production, consumable insects are a promising alternative food source. Furthermore, it is now widely accepted that eating insects can have positive health effects and that, when prepared in specific ways or cooked, eating insects poses no safety risks	Aguilar‐Toalá et al. (2022)
Most common edible insects	All stages of edible insects	USA	The objective of this review was to offer a concise overview of findings from human intervention studies that investigate the impacts of entomophagy on human well‐being	According to this review's findings, insects can (1) increase enlargement and modify iron state once added to complementary diets; (2) amend gut microbiota by means of some prebiotic impact; and (3) offer amino acids like soy protein. Also, in insect–herb mixture used in conjunction with standard medication may help to reduce the signs and signs of chronic disruptive pulmonary disease	Stull (2021)
Most common edible insects	Every phase of consumable insects	South Africa	Evaluating local people practical know how systems of insect production, consumption, policy, and sustainability	The conclusions of this review study suggested that interested parties, including local people and nationwide governments, be able to utilize them to make extra informed choices on the input of local knowledge for the production and sustainability of edible insects. The year‐round availability of insect production could be achieved by developing improved conservation techniques	Selaledi et al. (2021)
Common edible insects	All stages of edible insects	Poland	To evaluate and identify any potential threats, knowledge gaps regarding breeding, and possible factors for the bringing insects to the European market that may exist	“Significant knowledge gaps surrounding breeding practices, processing raw materials, and health safety were also found in the report, as were the risks that undoubtedly contribute to European customers' mistrust and decreased acceptability”	Skotnicka et al. (2021)
Common edible insects	All stages of edible insects	Finland	This study investigates how insect protein can help Europe's food intake reduce its propensity to cause global warming	This review paper's findings showed that, when compared to other sources of protein, consuming insects as food directly has the highest tendency to reduce consumers' carbon footprints in Europe. Second, they contend that using insects as animal feed can greatly reduce the potential for global warming in broiler production systems, in addition to the fact that low‐value side streams are necessary for optimizing this potential	Vauterin et al. (2021)
Mealworm	Adult	Netherlands	To evaluate how reducing the intake of milk and mealworm protein impacts the process of amino acid absorption kinetics, the rates of skeletal muscle protein synthesis after a meal, and the utilization of amino acids from dietary protein in human muscle protein during periods of rest and post‐exercise recovery.	This research finding indicate that ingesting a protein quantity resembling a meal from smaller mealworms leads to rapid protein digestion and amino acid absorption, resulting in an elevated rate of muscle protein synthesis during both rest and post‐exercise recovery. Similarly, comparable quantities of milk protein concentrate consumed by humans and smaller mealworms have equivalent effects on post‐meal protein processing.	(Hermans et al. 2021)
Crickets, locusts, mealworms, Superworm, Silkworm, Waxworm, Termites, Palm worm, Sago grub, and grasshoppers	Larvae	Netherlands	The aim of this study was to evaluate how insects contribute to the intake of iron and zinc in the human diet	Elevated protein content in edible insect species is associated with increased concentrations of iron (Fe) and zinc (Zn). Notably, there exists a meaningful positive relationship between the levels of Fe and Zn. In the realm of biochemistry, insects primarily employ proteins such as ferritin, transferrin, and other transport and storage proteins as the primary binding sites for non‐haem forms of Fe and Zn	Mwangi et al. (2018)
Common fit for human consumption insects	All life cycle phases of consumable insects	Africa	Assessing the accessibility, diversity, commonalities, and utilization of insects in African diets and as animal feed	The survey indicates that Africa consumes more than 470 distinct insect species. Lepidoptera, Orthoptera, and Coleoptera stand out as the most consumed insect types on the continent. Central Africa remains the primary hub for entomophagy culture, and in Africa, the utilization of insects for both nourishment and animal feed presents a practical choice	Kelemu et al. (2015)
Termites (*Macrotermes subhylanus*)	Adult	Kenya	To develop new products baked food items made from wheat buns enriched with edible insects and assess the foodstuffs nutritive as well as sensory attributes	The protein, retinol, riboflavin, iron, and zinc levels saw a significant rise of 16%–53% after the 5% substitution. The positive reception of wheat‐termite buns with a 5% substitution by customers highlights the considerable potential of insects for large‐scale commercial production, aiming to enhance food security in Africa	Kinyuru et al. (2009)
Mealworms ( *Tenebrio molitor* )	Larvae	Germany	To investigate how different drying methods and processing elements affect the stability of nutrients and lipid oxidation	The solubility of the protein decreased during drying using a vacuum, fluidized bed, and microwave methods. Mealworms that had been frozen‐dried had the highest levels of oxidation compared to further dried mealworms. This research illustrates that using a vacuum oven or microwave to dry mealworms can serve as a viable alternative to the traditional freeze‐drying method	Kröncke et al. (2018)
Mealworms ( *Tenebrio molitor* )	Larvae	Belgium	To evaluate if microwave drying can serve as a viable substitute for freeze drying in the context of mealworms, examining its impact on nutritional quality and color	The proximate composition of mealworms only differed slightly among drying methods, and microwave drying was the one to decrease the vitamin B12 concentration. Compared to the fat of mealworms that were microwave‐dried, the fat ratio from freeze‐dried mealworms displayed a greater level of oxidation	Lenaerts et al. (2018)
Mealworm ( *Tenebrio molitor* L.)	Larvae	Germany	The objective of this research was to explore the impact of different pre‐treatment and drying techniques on the physicochemical attributes and dry fractionation tendencies of mealworms	The study's findings showed that pretreatment and drying techniques affected the physicochemical characteristics of mealworms. In addition to offering an exciting technique to reduce the amount of water and energy consumed during the insect fractionation process, dry fractionation approaches have demonstrated the potential to produce a variety of mealworm fractions with various protein contents	Purschke et al. (2018)
House cricket, field crickets, mealworm, superworm, waxworm, silkworm, mopane caterpillar	Larvae and adults	Poland	The objective of this study was to assess and compare the nutritional content of edible insects with that of conventional meat	The study's results indicated that both meat and insects are rich sources of nutrients crucial for maintaining a healthy human body. However, there is a notable variation in the levels of these nutrients between meat and insects	Orkusz (2021)
Mealworms, Coleoptera, crickets, and locusts, Orthoptera	Larvae and adults	Poland	To investigate how heat treatment affects the antioxidant and anti‐inflammatory characteristics of peptides generated during simulated gastrointestinal digestion and absorption in a laboratory setting	The results of this investigation showcased that edible insect exhibited substantial antiradical activity, iron ion chelation capability, and the potential to inhibit lipoxygenase and cyclooxygenase‐2 activities during the simulated digestion and absorption phases. These outcomes underscore the notable influence of heat treatment on protein accessibility for enzymatic digestion, leading to an augmentation in the content of antioxidant peptides	Zielińska et al. (2017)
12 edible insects and 2 other invertebrates	—	Italy	To investigate whether extracts derived from 12 commercially accessible edible insects and two additional water‐ and fat‐soluble invertebrates' exhibit in vitro antioxidant properties	The findings from this research indicated that edible insects and invertebrates could potentially serve as a novel source of undiscovered redox compounds. These substances have a minimal ecological footprint and offer substantial antioxidant effectiveness, which is linked to their specific taxonomy and dietary patterns	Di Mattia et al. (2019)
Cricket ( *Gryllodes sigillatus* )	—	USA	To examine how enzymatic hydrolysis can affect the bioactive qualities and allergenicity of cricket	In the unhydrolyzed cricket and the cricket protein hydrolysates (CPH) with 15%–50% degree of hydrolysis (DH), all sera favorably reacted to tropomyosin, but no reactivity was seen at 60%–85% degree of hydrolysis. Consequently, when compared to other CPH and the unhydrolyzed control, CPH with a DH of 60%–85% displayed the most significant bioactive potential while demonstrating the least reactivity to tropomyosin	Hall et al. (2018)
*T. molitor* and *U. dermestoides*	Adult	Mexico	The aim of this study was a comparison of two edible adult insects' nutritional profiles, antioxidant capacities, and antimicrobial capacities	As per the findings of this study, *Tenebrio molitor* and Ulomoides dermestoides emerge as promising sources of both fiber and protein. Additionally, their antioxidant properties render them suitable candidates for use in nutraceutical food products	Flores et al. (2020)
Yellow mealworm ( *Tenebrio molitor* )	Larvae	China	The aim of this research was to assess the immunomodulatory capacity of extracts derived from the freeze‐dried powder of yellow mealworm ( *Tenebrio molitor* ) using supercritical carbon dioxide (CO_2_)	The findings of this research indicated that the extraction of freeze‐dried *Tenebrio molitor* larvae powder using supercritical CO_2_ fluid (fdTML) has the potential to serve as a dietary supplement	Tang and Dai (2016)
Grasshopperand honeybee	Adult larvae and pupae	Israel	The purpose of this work was to extract, characterize, and assess the functional qualities of soluble proteins from honeybees and edible grasshoppers	The findings of this study suggested that honeybees and grasshoppers do have the tendency for upcoming use as human diet, livestock feed, or insect‐based complimentary diet	Mishyna et al. (2018)
Stink bugs ( *E. delegorguei* )	Adult	Zimbabwe	To evaluate how traditional postharvest handling and storage conditions can affect the nutritional profile and mycotoxin accumulation in edible stink bugs	The findings of this study demonstrated that edible stink bugs are a nutrient‐ and antioxidant‐rich dietary source with positive effects on human health. As a result, using improved handling and storage techniques can help prevent the carcinogen aflatoxin from contaminating edible stink bugs, ensuring their safety as food for humans	Musundire et al. (2016)
Widespread consumable insect species	Every developmental phase of edible insects	Italy	The aim of this study was to evaluate how consumers perceive and understand edible insects	The study results offered crucial information for policymakers and consumers who are thinking about using food alternatives. To develop a market for food made from insects, the right consumers must be targeted. By educating people about the advantages of eating insects, this research helps to solve issues of global food security and sustainability	Simeone and Scarpato (2022)
Common consumable nsects	The entire stages of edible insects	China	This review paper aims to present a comprehensive summary of the nutritional benefits, health implications, and real‐world uses of consumable insects	The biological processes, nutritional composition, and safety of consumable insects in diet and medicine are all examined in this article. Because of their greater protein, amino acid, lipid, vitamin, and mineral composition, consumable insects make an exceptional resource of additional nutrition for human's diet	Zhou et al. (2022)

### 5.1 Biological Activities of Edible Insects

These qualities imply that these bioactive substances may be promising for enhancing human health (Aguilar‐Toalá et al. 2022). However, there is a lack of human clinical trials to conclusively establish their diverse bioactivities and potential health benefits, particularly in the context of preventing and managing chronic diseases. Therefore, more research is required to identify and characterize the insect component(s) responsible for these bioactivities using high‐efficiency and high‐resolution techniques, combined with carefully planned and controlled clinical intervention studies (Aguilar‐Toalá et al. 2022). Hence, edible insects can serve as components in nutraceuticals and functional food products and are recognized as a valuable nutrient source for human consumption (Patel et al. 2019).

## 6 Edible Insects Safety

Presently, there is a significant level of apprehension among consumers regarding the safety of consuming edible insects, especially in nations with a lower economic status. These regions consider edible insects as integral components of their culture, culinary traditions, and dietary staples (Imathiu 2020; Baiano 2020). In addition to the nutrient profile, additional research should be conducted to determine whether potentially dangerous components, including toxins, allergies, and antinutrients, are present. Decontamination techniques and storage procedures must be assessed and developed because of potential microbiological hazards and the rapid deterioration of raw edible insects (Rumpold and Schl 2013). The most frequent safety concerns for edible insects are their anti‐nutritional and microbiological qualities, pesticides, heavy metals, mycotoxins, and allergies (Aguilar‐Toalá et al. 2022).

Legal and food safety challenges related to the consumption of edible insects exist in the European Union (EU). Within the EU, uncertainty remains regarding the classification of insect‐based products as novel foods, which are defined as those not traditionally consumed to a significant extent (Van der Spiegel et al. 2013). Consequently, it is imperative to advocate for the enforcement of food safety and hygiene standards across the entire edible insect supply chain, including the wild harvesting phase. This ensures that this exceptionally nutritious food source, which requires minimal resources for production, is accessible to consumers without posing health hazards. Another issue that requires urgent attention is the lack of rules on the value chain for edible insects in many nations, particularly developing nations. Addressing this issue is crucial for increasing customer confidence and facilitating the trading of edible insects (Imathiu 2020).

### 6.1 Anti‐Nutritional Factors of Edible Insects

Edible insects are now recognized as sustainable protein‐rich substitutes for traditional animal protein sources. Anti‐nutritive factors impair gastrointestinal and metabolic functions by reducing nutrient utilization or food intake (Meyer‐Rochow et al. 2021). However, concerns about their overall nutritional quality have arisen because of the presence of anti‐nutritional factors (ANFs) such as tannins, phytic acid, oxalate, and chitin in certain insect species (Ojha, Bekhit, et al. 2021; Sailo et al. 2020). One prominent ANF found in edible insects is chitin, a complex polysaccharide that forms a structural component of their exoskeletons (Kipkoech 2023). While chitin itself is not harmful, its presence in significant amounts can reduce the bioavailability of essential nutrients, such as proteins and minerals (Kipkoech 2023).

Challenges associated with chitin include its interference with the digestion process, which limits access to valuable nutrients in insect biomass (Rodríguez‐Rodríguez et al. 2022). The impact of ANFs on nutritional absorption necessitates strategic processing and consumption approaches. Various methods, including grinding, cooking, and enzymatic treatments, have shown promise for reducing chitin content, enhancing the digestibility of insect‐based products, and improving nutrient absorption (Zhou et al. 2022; Mohd Zaini et al. 2023; Lee et al. 2021).

Research efforts are ongoing to identify insect species with inherently low levels of ANFs and to develop more efficient processing techniques. Addressing the issue of ANFs is crucial for the successful integration of edible insects into mainstream diets, ensuring that these alternative protein sources not only offer sustainability benefits but also meet nutritional expectations (Nyangena et al. 2020; Liceaga 2021; Manditsera et al. 2019; Schwarz and Zarei 2023). Therefore, the presence of anti‐nutritional factors in edible insects poses challenges, and ongoing research and advancements in processing techniques hold promise for optimizing their nutritional profile and promoting their widespread acceptance as valuable food sources.

### 6.2 Edible Insect Allergens

Edible insects are a promising and sustainable protein source; however, concerns about allergens must be addressed (Fernandez‐Cassi et al. 2018). Common allergenic proteins in insects, including tropomyosin, arginine kinase, and myosin, can trigger allergic reactions, particularly in individuals with insect allergies. Cross‐reactivity with crustacean shellfish allergies is also a potential risk, underscoring the importance of careful consideration of individuals with pre‐existing allergies (De Marchi et al. 2021; de Gier and Verhoeckx 2018; Ribeiro et al. 2018; Ribeiro et al. 2021).

Research indicates that certain edible insects may contain allergens that pose risks comparable to those of known food allergens (Ribeiro et al. 2021). It is crucial to raise awareness of these potential allergens and establish clear labeling practices to protect consumers, especially those with allergies (Allen et al. 2014). Additionally, exploring insect protein sources with lower allergenicity profiles or employing advanced processing techniques to reduce allergen content can improve the safety of edible insects (Liceaga 2022).

As the edible insect industry continues to grow, regulatory bodies must establish and enforce standards for allergen labeling and safety assessments (Żuk‐Gołaszewska et al. 2022). Manufacturers should implement rigorous quality control measures at every stage of production, from farming to processing, to minimize the risk of allergen contamination (Niassy et al. 2022). Overall, while edible insects offer a sustainable food solution, addressing allergen concerns is paramount to ensuring the inclusivity and safety of these novel protein sources for diverse consumer bases (Imathiu 2020).

### 6.3 Edible Insect's Biological Contaminants

Edible insects have gained prominence as a sustainable and protein‐rich food source; however, an in‐depth examination of potential biological contaminants, such as mycotoxins, pathogenic microorganisms, and fungi, is imperative to ensure their safety for human consumption (Evans and Shao 2022). Mycotoxins produced by molds are a significant concern in edible insect farming. These toxic compounds can infiltrate insect feed, leading to their accumulation in the insect biomass (Schrögel and Wätjen 2019). Addressing the sources of mycotoxin contamination is crucial for preventing adverse health effects. Establishing stringent quality control measures throughout the insect farming process, from feed production to the final product, is essential for mitigating the risks associated with mycotoxins (Schrögel and Wätjen 2019; Gałęcki et al. 2023).

Pathogenic microorganisms, including bacteria and viruses, pose potential health risks to consumers if they are present in edible insects (Fernandez‐Cassi et al. 2018). Maintaining high levels of hygiene in insect farms, implementing regular health monitoring of insect colonies, and adopting effective sanitation practices are critical for minimizing the spread of pathogenic microorganisms. Stringent protocols should be in place to ensure that insects destined for human consumption are free of harmful pathogens (Gałęcki et al. 2023; van Huis et al. 2013). Fungal contamination is another factor that affects the safety of edible insects. While some fungi contribute positively to the nutritional composition of insects, others may produce mycotoxins or adversely affect their quality (Evans and Shao 2022). Striking the balance in the microbial ecosystem within insect habitats and feed is vital for minimizing the risks associated with harmful fungi. A combined and varied population of microbes lives inside the exoskeletons, mouthparts, and intestines of insects (Douglas 2015). This requires careful management and monitoring of the environmental conditions during insect farming (Stathas et al. 2023).

To address these concerns, it is imperative that the edible insect industry establish and adhere to robust regulatory frameworks and industry standards. Rigorous testing of mycotoxins, pathogenic microorganisms, and fungi at different stages of production is essential to ensure the safety of the final product (Żuk‐Gołaszewska et al. 2022). Collaboration among researchers, farmers, and regulatory bodies is crucial for advancing our understanding of these contaminants and developing effective strategies to mitigate their impact on the safety and quality of edible insects (Niassy et al. 2022). In conclusion, addressing biological contaminants in edible insects requires a comprehensive approach involving strict quality control measures, continuous monitoring of mycotoxins and pathogenic microorganisms, and the effective management of fungal contamination. Industry‐wide standards and regulatory frameworks are essential for ensuring the safety and reliability of edible insects as sustainable and innovative protein sources in the global food supply chain.

### 6.4 Edible Insect Pesticide Residue

Edible insects, touted for their sustainability and nutritional benefits, face concerns regarding pesticide residues. The presence of residues originating from insecticides, herbicides, and fungicides used in farming poses risks to both human health and environmental sustainability (Hernández‐León et al. 2022; Labu et al. 2022). The diversity of pesticides raises questions regarding specific residues in edible insects, prompting the need for rigorous testing and regulatory measures at various production stages (Imathiu 2020). Adverse effects on human health, such as neurological and endocrine disruptions and potential ecological consequences, underscore the importance of addressing pesticide residues in edible insects. Promoting organic farming practices, integrated pest management, and sustainable alternatives are crucial for minimizing residue levels (Aguilar‐Toalá et al. 2022; Hernández‐León et al. 2022; Ali et al. 2021). Collaboration between stakeholders, including researchers, farmers, and regulatory bodies, is essential for establishing maximum residue limits and standardized testing protocols. In conclusion, while edible insects hold promise as sustainable protein sources, addressing pesticide residue concerns is pivotal for ensuring their safety and acceptance in the global food supply chain (Labu et al. 2022; van Huis et al. 2013; FAO 2022).

### 6.5 Heavy Metals in Edible Insects

The presence of heavy metals in edible insects is a scientific concern with implications for both human health and environmental sustainability (Malematja et al. 2023; Fernandez‐Cassi et al. 2018). Heavy metals, such as lead, cadmium, and mercury, can accumulate in insects from various sources, including their living environment and the feed they consume (Truzzi et al. 2019; Meyer et al. 2021; Fernandez‐Cassi et al. 2018). Studies have demonstrated the potential for adverse health effects in humans upon the ingestion of edible insects contaminated with elevated levels of these heavy metals. Neurological, developmental, and systemic effects are among the health risks associated with heavy metal exposure (Schrögel and Wätjen 2019).

To address these concerns, scientific research has emphasized the importance of rigorous monitoring and testing protocols to assess heavy metal concentrations in edible insects. Implementing and enforcing stringent regulatory frameworks is essential to establish safety standards for permissible levels of heavy metals in edible insect products (Niassy et al. 2022). Understanding the environmental impact of heavy metal accumulation in insects and their surroundings is crucial for the overall sustainability of insect farming as a protein source (Malematja et al. 2023). Collaborative efforts between scientists, policymakers, and industry stakeholders are essential to advancing our understanding, ensuring food safety, and establishing guidelines for sustainable insect production (Kelemu et al. 2015; Moruzzo et al. 2021; Niassy et al. 2022; Murefu et al. 2019).

## 7 Public Perception Toward Edible Insects

A growing number of people are turning to consumable insects as a substitute food resource in response to shifts in global climate patterns and increasing carbon emissions and ecological impacts associated with traditional farming methods. The advantages of edible insects as food include speedy reproduction, superior energy exchange efficiency, broad delivery, diversity, low greenhouse gas along with ammonia emissions, the chance to minimize waste, and high nutritional constituents (Musundire et al. 2021). Persuading a culture with reservations or aversions toward insects to adopt them as reliable food sources can be challenging.

The reason is not that Western individuals who avoid insects as a food source experience hunger. Rather, the widespread adoption of intensive farming methods influenced by Western philosophies could potentially lead to the depletion of wild foods, such as insects. This depletion might occur because of a lack of awareness regarding the vital role of insects in maintaining healthy ecosystems and their significance in food supply, as highlighted by Bharucha and Pretty (2010). The future of the insect industry in Western cultures is not favorable, despite the many benefits of eating insects. As consumers become more aware of the benefits of eating insects, they are more prepared to pay for insect food (Piha et al. 2016; Ramos‐Elorduy 1997; Defoliart 1999).

Continuous exposure‐building campaigns, along with advancements in taste and appearance, have successfully changed certain Western nations' unfavorable attitudes (Looy et al. 2014; van Huis et al. 2013). Customers in Belgium are increasingly accepting insects as a superior food source (Van Thielen et al. 2018). The Netherlands has effectively promoted the use of freeze‐dried insect powder as a viable alternative to meat, as highlighted by Raheem et al. (2018). To promote acceptance, consumers need to be reminded of the social, practical, and contextual elements that influence food consumption (House 2016).

Continuous efforts to educate and promote the use of edible insects underscore their potential to tackle current and future challenges related to the environment, population, and land availability. Existing research provides evidence of the substantial nutritional and healing advantages associated with insect consumption. The market for edible insects has expanded through a variety of tactics, and opposition to entomophagy in Western societies has also been combated (Kim et al. 2019). To successfully develop the worldwide market, Western perceptions toward edible insects must be modified.

Several factors contribute to the absence of entomophagy (the practice of eating insects) in Western societies, including insect size, scattered distribution, and seasonal unavailability (van Huis 2016). Moreover, even though only 0.2% of insects truly pose a threat to human life, persistent misconceptions about their danger contribute to people's resistance to entomophagy (van Huis 2016). The government, academia, industry, and research institutions have made significant efforts to change cultural and religious attitudes toward edible insect consumption.

## 8 The Environmental and Ecological Benefits of Entomophagy

Entomophagy, the practice of incorporating insects into the human diet, has attracted significant attention in recent years. This surge of interest is fueled by the potential to address critical sustainability and environmental concerns, particularly when compared to conventional livestock farming methods. Notably, researchers (Rumpold and Schl 2013; Lange and Nakamura 2021; FAO 2013; van Huis 2015) have recognized the significance of entomophagy as an innovative and sustainable source of protein, offering a promising solution to the environmental challenges associated with traditional meat production.

This comprehensive review undertakes a meticulous examination of the environmental aspects of entomophagy, with specific emphasis on recent studies (Tang et al. 2019; Payne et al. 2016). The primary objective was to evaluate whether entomophagy can serve as an eco‐friendly dietary alternative. Entomophagy has emerged as a potential solution in an era marked by growing concerns about the ecological impact of dietary choices (Oonincx et al. 2010; Matandirotya et al. 2022; Stull 2021; Raheem et al. 2018; Eilenberg and van Loon 2018). This review focuses on the most recent research and discoveries concerning entomophagy, considering its ecological, nutritional, and economic implications (Moruzzo et al. 2021; van Huis et al. 2021; (Payne et al. 2023).

Numerous studies have demonstrated the multifaceted environmental and ecological advantages of entomophagy. First, recent research (Lange and Nakamura 2023; van Huis and Oonincx 2017; Lumanlan et al. 2022; Lin et al. 2023; Tao and Li 2018; Smith et al. 2021) has underscored the impressive resource efficiency of insect farming in comparison to traditional livestock agriculture. Insects demand significantly less water, land, and feed, leading to a notable reduction in the carbon footprint of food production (van Huis and Oonincx 2017; Smith et al. 2021). These findings emphasize the potential of insect farming to alleviate pressing environmental concerns, including water scarcity and greenhouse gas emissions (Lange and Nakamura 2023). Moreover, the preservation of biodiversity in the context of agriculture is a critical issue (Lange and Nakamura 2023; Muluneh 2021), and investigations by Lin et al. (2023) have investigated the potential impacts of insect farming on ecosystems. While insect farming is not without challenges, it can mitigate habitat destruction often linked to conventional agriculture and, in some instances, even foster biodiversity conservation (Jankielsohn 2023; Busse et al. 2021).

### 8.1 Edible Insects as for Sustainable Waste Reduction and Circular Economy in Food Production

Entomophagy, the consumption of insects, has emerged as a promising solution for addressing the dual challenges of waste reduction and sustainable food production. This practice aligns with the principles of a circular economy, emphasizing the efficient use of resources and minimizing environmental impact (Lange and Nakamura 2023; Ojha et al. 2020; Moruzzo et al. 2021). Recent research has highlighted the remarkable ability of insects to convert organic waste into valuable proteins, presenting an innovative approach to minimize waste and alleviate the environmental burden associated with conventional food production methods (Kim et al. 2021; Ojha et al. 2020; Lin et al. 2023; Mannaa et al. 2024; Salomone et al. 2017).

In addition, the potential health benefits of entomophagy have gained attention in recent years (Nowakowski et al. 2022). Research by Tang et al. (2019) and Lange and Nakamura (2023) suggested that insects can serve as a rich source of proteins, vitamins, and minerals, while reducing their reliance on harmful pesticides and chemicals in food production (Imathiu 2020), thereby contributing to healthier (Aguilar‐Toalá et al. 2022) and more environmentally friendly food systems (Aidoo et al. 2023; Imathiu 2020). However, entomophagy faces certain challenges including regulatory hurdles and the need for consumer acceptance. While the environmental benefits of insect farming are substantial, overcoming regulatory barriers and shifting public perception remain significant challenges to be addressed to promote insect farming as a sustainable food source (Żuk‐Gołaszewska et al. 2022; Lähteenmäki‐Uutela et al. 2021; Aidoo et al. 2023).

### 8.2 The Role of Edible Insects in Pollination and Agriculture for Eco‐Benefits and Sustainability

When examining the potential of insects and their environmental and ecological benefits in plant pollination and supporting agriculture, it is evident that insects play a crucial role in sustainable farming practices (Kjohl et al. 2011). The intricate interplay between insects and plants underscores their significance in maintaining ecological balance and fostering agricultural productivity (Aidoo et al. 2023; Siddiqui et al. 2022; Moruzzo et al. 2021).

Insects, such as bees and specific beetle species, are pivotal in plant pollination. Their roles go beyond merely transferring pollen; these pollinators contribute significantly to increasing crop yields and ensuring the overall health of ecosystems (Hristov et al. 2020; Silva et al. 2020). Through efficient pollination activities, insects promote genetic diversity in plant populations, foster resilience against environmental stressors, and enhance the adaptability of crops to changing conditions (Aizen et al. 2017).

Furthermore, the environmental benefits of insect‐rearing for plant pollination extend to the reduction of reliance on chemical pollinators (Gabriel and Tscharntke 2007). Unlike synthetic alternatives, insects engage in natural and sustainable processes that do not introduce harmful substances into the environment (Kjohl et al. 2011). This shift toward biological pollination methods aligns with eco‐friendly agricultural practices, minimizing the ecological footprint associated with conventional farming (Sawe et al. 2020).

In addition to their role in pollination, insects also contribute significantly to supporting agriculture through various mechanisms (Kjohl et al. 2011). Efficient recycling of organic waste by certain insect species presents an eco‐friendly solution for waste management in agricultural settings. Insects adept at converting organic matter into valuable proteins not only minimize waste but also contribute to the creation of nutrient‐rich soil, enhancing overall soil health and fertility (Salam et al. 2023; Mannaa et al. 2024).

The benefits of insect‐rearing for agriculture extend to the conservation of biodiversity. By adopting insect‐friendly practices, farmers can create habitats that support a diverse range of species, including those crucial for natural pest control (Duffus et al. 2023). This integrated approach reduces the need for chemical pesticides and promotes a more balanced and sustainable agricultural ecosystem (Köthe et al. 2023).

Despite these positive aspects, challenges remain in the effective management and integration of insects into agricultural practices. Addressing issues related to optimal insect management, ensuring compatibility with existing farming methods, and navigating regulatory frameworks are critical for maximizing the potential for insect rearing in agriculture (van der Sluijs and Vaage 2016; Lange and Nakamura 2023; Aidoo et al. 2023; Lumanlan et al. 2022).

In conclusion, the potential for insects and the environmental and ecological benefits of rearing for plant pollination and supporting agriculture are multifaceted and promising. Insects offer a sustainable and eco‐friendly approach to modern agriculture by enhancing crop yields through natural pollination to contribute to waste recycling and biodiversity conservation. As we strive for more sustainable food production systems, understanding and harnessing the potential of insects in agriculture is becoming increasingly important for a resilient and environmentally conscious future.

## 9 Conclusion

In summary, edible insects present a promising solution to global protein requirements because of their exceptional nutritional value and multifaceted benefits for the economy and environment. They offer a sustainable and nutritious protein source that is rich in proteins, essential amino acids, omega‐3 fatty acids, vitamins, and minerals. The nutritional composition of edible insects is influenced by the substrate or material from which they are reared. This alternative protein source can significantly enhance long‐term food and nutritional security. Their consumption aligns with the growing demand for sustainable food production because insects require fewer resources and emit fewer greenhouse gases than traditional livestock. Moreover, edible insects play a crucial role as pollinators in ecosystems, contributing to biodiversity and supporting agriculture. This dual role underscores their significance in a changing world, which requires a balance between food needs and environmental preservation. However, challenges related to safety and health exist in the consumption of edible insects, although research has suggested that they are reliable sources of bioactive substances with potential health benefits. To fully exploit the potential of edible insects, the food industry must create appetizing insect‐based products for consumer acceptance. Additionally, integrating insects into our diets, potentially replacing traditional protein sources, such as red meat and poultry, needs to become more widespread. In conclusion, edible insects offer a sustainable, nutritious, and environmentally friendly solution to global protein requirements, addressing pressing food‐related challenges while preserving the health and biodiversity of the planet.

## Author Contributions

**Belay Gezahegn Gebreyes:** conceptualization (equal), visualization (equal), writing – original draft (equal), writing – review and editing (equal). **Tilahun A. Teka:** conceptualization, editing, revision, and methodology.

## Conflicts of Interest

The authors declare no conflicts of interest.

## Data Availability

This manuscript constitutes a comprehensive review paper, wherein all essential information and data have been meticulously sourced from the scientific references cited throughout the text.
