# Correction to “The Environmental and Ecological Benefits of Edible Insects: A Review”

**DOI:** 10.1002/fsn3.70753

**Published:** 2025-07-28

Gebreyes B. G. Teka T. A. 2025 “The Environmental and Ecological Benefits of Edible Insects: A Review.” *Food Science & Nutrition* 13, no. 6: e70459. https://doi.org/10.1002/fsn3.70459.

We would like to clarify that the second author, Tilahun A. Teka, is affiliated with the Department of Postharvest Management, College of Agriculture and Veterinary Medicine, Jimma University, Jimma, Ethiopia. However, during the revision and proofreading process, we mistakenly listed his affiliation as the Ethiopian Institute of Agricultural Research, Teppi Agricultural Research Center, Teppi, Ethiopia.

The correct affiliations are:

Belay Gezahegn Gebreyes^1^˒^2^, Tilahun A. Teka^1^

^1^Department of Postharvest Management, College of Agriculture and Veterinary Medicine, Jimma University, Jimma, Ethiopia

^2^Ethiopian Institute of Agricultural Research, Teppi Agricultural Research Center, Teppi, Ethiopia

We sincerely apologize for this error.

